# Optically stimulated luminescence in state-of-the-art LYSO:Ce scintillators enables high spatial resolution 3D dose imaging

**DOI:** 10.1038/s41598-022-12255-9

**Published:** 2022-05-18

**Authors:** Mads L. Jensen, Jacob S. Nyemann, Ludvig P. Muren, Brian Julsgaard, Peter Balling, Rosana M. Turtos

**Affiliations:** 1 Department of Physics and Astronomy, Aarhus University, Ny Munkegade 120, 8000 Aarhus, Denmark; 2 Danish Centre for Particle Therapy, Aarhus University Hospital, Palle Juul-Jensens Boulevard 99, 8200 Aarhus, Denmark; 3 Department of Clinical Medicine, Aarhus University, Palle Juul-Jensens Boulevard 82, 8200 Aarhus N, Denmark

**Keywords:** Materials science, Imaging techniques, Imaging techniques, Radiotherapy, Characterization and analytical techniques, Optical physics, Techniques and instrumentation

## Abstract

In this contribution, we study the optically stimulated luminescence (OSL) exhibited by commercial $\hbox {Lu}_{(2-x)}\hbox {Y}_x\hbox {SiO}_5$:Ce crystals. This photon emission mechanism, complementary to scintillation, can trap a fraction of radiation energy deposited in the material and provides sufficient signal to develop a novel post-irradiation 3D dose readout. We characterize the OSL emission through spectrally and temporally resolved measurements and monitor the dose linearity response over a broad range. The measurements show that the $\hbox {Ce}^{3+}$ centers responsible for scintillation also function as recombination centers for the OSL mechanism. The capture to OSL-active traps competes with scintillation originating from the direct non-radiative energy transfer to the luminescent centers. An OSL response on the order of 100 ph/MeV is estimated. We demonstrate the imaging capabilities provided by such an OSL photon yield using a proof-of-concept optical readout method. A 0.1 $\hbox {mm}^3$ spatial resolution for doses as low as 0.5 Gy is projected using a cubic crystal to image volumetric dose profiles. While OSL degrades the intrinsic scintillating performance by reducing the number of scintillation photons emitted following the passage of ionizing radiation, it can encode highly resolved spatial information of the interaction point of the particle. This feature combines ionizing radiation spectroscopy and 3D reusable dose imaging in a single material.

## Introduction

Scintillation is found in a wide variety of both organic and inorganic materials and is defined as the efficient conversion of incident ionizing particles into an optical response of fast rise and/or decay times. This ability has made scintillators the cornerstone of room temperature ionizing radiation spectroscopy and imaging. A high-performing family of scintillators is the cerium-doped lutetium orthosilicate $\hbox {Lu}_{(2-x)}\hbox {Y}_x\hbox {SiO}_5$:Ce (LYSO:Ce) crystals developed in the ’90s, which have an exceptional high photon-emission rate. This material has enabled the development of the current generation of time-of-flight positron emission tomography (TOF-PET) clinical scanners and is of high interest for e.g. reconstruction-free TOF-PET imaging and online monitoring of adaptive proton therapy^1,2^ enabled by its high photon-emission rate.

**Figure 1:**
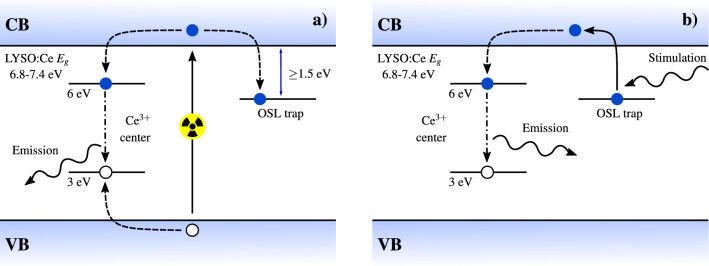
Band gap diagram for LYSO:Ce. (**a**) illustrates the scintillation process: Ionizing radiation generates electrons and holes, which are subsequently trapped by $\hbox {Ce}^{3+}$ centers with a life time of $\sim \; 40$ ns and give rise to prompt light emission. A fraction of electrons are stored in auxiliary OSL traps, which are readout on request [see panel (**b**)] through the same $\hbox {Ce}^{3+}$ centers using optical stimulation. Solid lines illustrate excitations, dashed lines illustrate capture processes, dash-dotted lines illustrate radiative decays and wavy lines illustrate optical photons.

Figure 1a illustrates the scintillation process within the band gap of such LYSO:Ce crystals. Upon interaction with ionizing radiation, electrons are excited across the band gap, where they generate secondary electrons and holes that thermalize to the lowest available energy in their respective bands. Energy from the thermalized charge carriers is transferred to the $\hbox {Ce}^{3+}$ centers energetically located within the 6.8–7.4 eV band-gap^3^ of the insulating inorganic matrix. This capture has a characteristic time ranging from 50 to 200 ps under X-ray or gamma excitation and explains the rise time of the scintillation signal which has been reported as non-mono-exponential^4^. The $\hbox {Ce}^{3+}$ centers function as recombination centers through a 5d-4f optical transition characterized by a decay time of $\sim \; 40$ ns, and are responsible for the scintillation in this material, producing around 40,000 optical photons per deposited MeV^5^. As illustrated in Fig. 1a, capture to another more long-lived trap state also occurs, allowing for storage of a fraction of the excited electrons.

Electrons captured and trapped in this metastable trap state can be excited post-irradiation using low-energy optical stimulation allowing for radiative recombination through the $\hbox {Ce}^{3+}$ centers as illustrated in Fig. 1b. Such optically stimulated luminescence (OSL) provides a method to interrogate the OSL-traps and assess the number of trapped electrons, as this correlates directly with the number of emitted OSL-photons^6^. Notably, the energy of the emitted OSL-photons can be higher than that of the stimulating photons without violating energy conservation, which enables efficient filtering of any long-wavelength photoluminescence. Previous studies^7–9^ of lutetium orthosilicate (LYSO) compounds using films, powders, and structurally defective cerium doped single crystals have reported OSL properties in these compounds. These studies agree on the stability and dose-linearity of OSL in the investigated LYSO compounds and the relevance of this energy-storage mechanism in radiation dosimetry. Oxygen vacancies have previously been identified as electron traps in cerium-doped LYSO crystals and proposed as a candidate for these OSL-traps^9,10^. However, the study and application of OSL in commercial LYSO:Ce samples used for current TOF-PET imaging remains to be explored.

The spatial distribution of the energy deposited in the scintillator is reflected by the OSL-photons, which can be extracted using appropriate optical excitation and an imaging setup. As previously suggested^11–14^, this makes OSL an excellent photon emission candidate to develop a passive and reusable novel 3D dosimeter. Three-dimensional dosimetry is continuously being developed as an appropriate tool for validation and quantification of complex treatment plans in modern radiotherapy (RT). Currently, multidimensional dosimetry is either performed during irradiation with the use of several charge-coupled device (CCD) cameras and plastic/liquid scintillators^15–17^ or using a combination of water-phantoms and multiple detectors^18^, making real-time dose monitoring instrumentally heavy. Post-irradiation readout, or passive dosimetry, is led by gel or bulk radiochromic materials, where dose-correlated information is read out with an optical computed tomography scanner^19^. Recent applications have demonstrated dosimetry inside the magnetic field present in novel MR-guided radiotherapy^20^, while the high spatial resolution of radiochromic dosimeters has allowed imaging of, e.g., the localized dose (Bragg peak) in proton therapy^21^, and the demonstration of the possibility to use a flexible host matrix was used to investigate the effects of deformation during proton therapy^22^. Nonetheless, the existing 3D dosimeters are based on materials with an irreversible chemically induced response to ionizing radiation, a response which has been shown to exhibit time- and temperature variations^23^, and they are by design one-time use dosimeters, which typically require batch-specific calibration. Hence, a reusable OSL-based post-irradiation dosimeter, given a precise readout method, could tackle the challenges associated with digital multidimensional dosimetry for quality assurance in RT.

The study and application of OSL in state-of-the-art commercial LYSO:Ce scintillators presented in this contribution is divided into two parts. First, we estimate the order of OSL photons emitted per energy deposited, i.e. OSL yield, in one of the fastest and brightest scintillators in use nowadays for TOF-PET imaging^24^. To our knowledge, this is the first time any study reports on the energy conversion efficiency of the OSL mechanism in these crystals. Second, we demonstrate a proof-of-concept 3D readout system capable of retrieving the stored dose information from the OSL traps along with an estimation of the obtainable signal-to-noise ratio (SNR) based on the OSL yield of LYSO:Ce and the properties of the detection system.

## Results

### Characterization of the OSL

**Figure 2:**
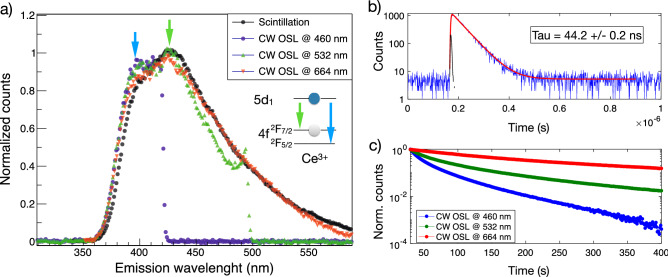
Optically stimulated luminescence mechanism in LYSO:Ce single crystals. (**a**) Spectrally resolved OSL excited with blue, green and red continuous-wave (CW) lasers in comparison to the LYSO:Ce scintillation spectrum. The sharp cut-offs observed for the blue and green stimulation are due to necessary filtering of laser light. (**b**) Decay curve from pulsed OSL (blue) fitted to a mono-exponential decay (red) with decay time ($44.2 \pm 0.2$) ns. The black curve shows the instrument response function (FWHM 6 ns). (**c**) CW-laser stimulated OSL decay curves obtained similar excitation powers with energies of 1.87, 2.33, and 2.70 eV, i.e red, green, and blue, respectively.

The OSL mechanism was investigated by irradiating an LYSO:Ce crystal with a $^{90}$Sr/$^{90}$Y-source, and stimulating the OSL post-irradiation using continuous-wave (CW) laser excitation with energies of 1.87, 2.33, and 2.70 eV, i.e. red, green, and blue laser diodes, respectively. The emitted light was imaged onto a spectrograph equipped with a low-noise CCD camera to allow for measurement of spectrally resolved OSL. The same spectrograph was used to assess the spectrally resolved scintillation emission via a fiber-coupled connection to the crystal during irradiation. As demonstrated in Fig. 2a, the scintillation and OSL show great similarity, as we observe all spectra to have the same rising edge and shape, characteristic of the $\hbox {Ce}^{3+}$ absorption edge and emission bands, respectively. Furthermore, we are also able to resolve the 5d–4$\hbox {f}_{5/2}$ and 5d–4$\hbox {f}_{7/2}$ transition of the cerium one center^25^ with emission wavelengths at 390 nm and 425 nm, respectively (marked by the blue and green arrow in the Fig. 2a).

We examined the kinetics of the OSL mechanism by pulsed stimulation using a femtosecond laser with 460 nm wavelength and employing a time-correlated single-photon counting readout. The results are shown in Fig. 2b, where a mono-exponential decay time of 44 ns characteristic of the $\hbox {Ce}^{3+}$ emission centers can be observed. For these measurements, we monitored the background by exciting the LYSO:Ce cube with the femtosecond laser while the OSL traps are empty, which confirms that the data presented is not due to the scintillation emission nor population of OSL traps from the intrinsic $^{176}$Lu radioactivity. The instrumental response function of the setup was obtained by directly measuring the laser light reflected at the face of the LYSO:Ce crystal.

The above findings support the model (Fig. 1), showing the OSL mechanism to have identical spectral characteristics and kinetics as the well-characterized $\hbox {Ce}^{3+}$ center responsible for scintillation.

The relative photoionization cross section of the OSL process for each of the three stimulating laser wavelengths was investigated using a photo-multiplier tube (PMT) in photon counting mode and appropriate filtering to isolate the OSL photons. Figure 2c displays the normalized CW OSL signal measured from an LYSO:Ce crystal irradiated 1 min under the $^{90}$Sr/$^{90}$Y-source and read out with the three stimulating laser wavelengths, all with similar spatial profiles and powers. These measurements indicate an increasing cross section for excitation with increasing photon energy, as the readout of the crystal reaches background levels significantly faster at higher energies. Hence, a blue laser will yield the fastest readout of occupied OSL traps.

We estimate the OSL yield by defining $\hbox {LY}_\mathrm {OSL} = N_\mathrm {emit}/E_\mathrm {dep}$, where $N_\mathrm {emit}$ is the total number of emitted OSL photons after deposition of energy $E_\mathrm {dep}$. Geant4 simulations^26^ were used to estimate the energy deposition rate in the crystal to $1.06 \times 10^9$ MeV/min during irradiation with the $^{90}$Sr/$^{90}$Y-source. The total number of emitted OSL photons is assessed by integrating the generated photoelectrons in the PMT over time, upon stimulation of the LYSO:Ce crystal with the blue 460 nm CW laser until a background level is reached. The total number of emitted photons is calculated as $N_\mathrm {emit}= N_\mathrm {pe}/\mathrm {PDE}$, where $N_\mathrm {pe}$ is the number of detected photoelectrons and $\mathrm {PDE} = \mathrm {QE}\cdot \mathrm {OCE}\cdot \mathrm {LTE}$ is the photon detection efficiency. Here, QE is the PMT quantum efficiency, which averages to around 25% for the wavelength range measured, and LTE describes the light transfer efficiency of the LYSO:Ce crystal, usually around 10% when the crystal readout face is air-coupled to the PMT, as previously reported^5^. This factor describes the fraction of the generated photons that escape through the readout face of the crystal, taking into account both internal reflection, absorption, and refraction at the interface. The OCE describes the optical coupling efficiency of the setup, which is affected by the lens, filters, and the distance between the PMT and the sample. This last factor has been measured using a methodology described in the “Methods” section and reaches values of around 1%. With all these factors taken into account and with a measured $N_\mathrm {pe} = 30$ million when irradiating the LYSO:Ce for one minute with a $^{90}$Sr/$^{90}$Y-source, we arrive at a final estimate of around 100 OSL photons/MeV with wavelengths between 330 and 420 nm corresponding to the used filter window. This result points towards a small branching ratio between capture to the OSL traps and capture to the $\hbox {Ce}^{3+}$ centers.

**Figure 3:**
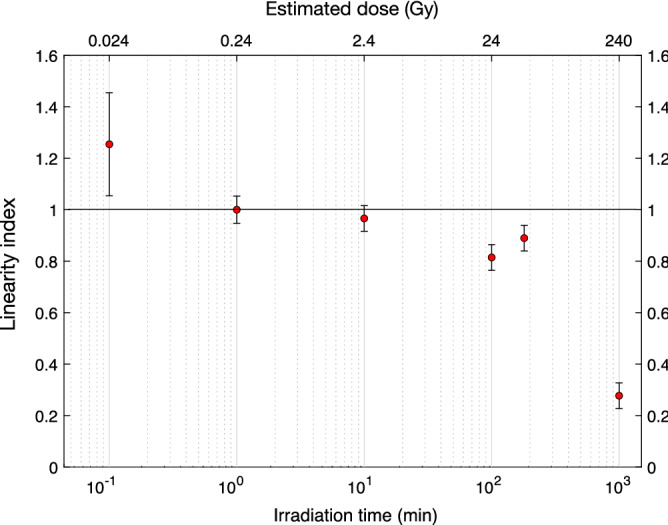
LYSO:Ce dose linearity response to $^{90}$Sr/$^{90}$Y electron excitation. OSL-response per dose at varying irradiation times plotted relative to the 1 min irradiation. The dose values shown in the top X-axis were estimated using Geant4-based Monte Carlo simulations, representing the average value within the outermost $10\times 10\times 1$ $\hbox {mm}^3$ layer facing the radiation source.

**Figure 4:**
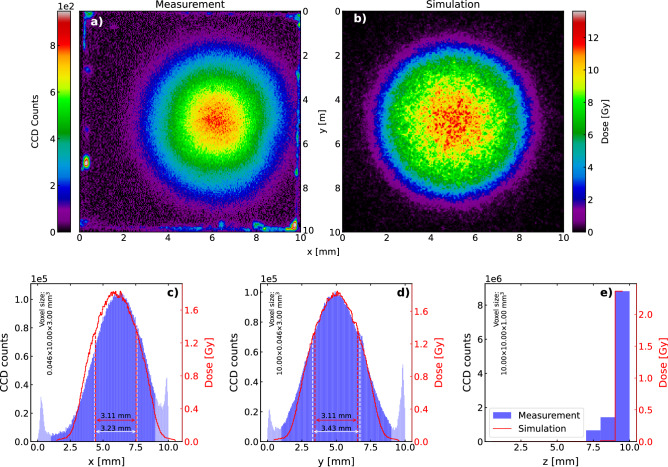
Spatially resolved post-irradiation OSL readout from a $^{90}$Sr/$^{90}$Y-irradiated LYSO:Ce crystal. The crystal was irradiated by a $^{90}$Sr/$^{90}$Y-source for 10 min. (**a**) Readout of the first mm of the crystal, as seen from the source. The voxel size is 0.046 $\times$ 0.046 $\times$ 1.00 $\hbox {mm}^3$. For display purposes, the color map is adjusted to saturate (gray) at very high count numbers and negative values are interpreted as background fluctuations and displayed as zero counts. (**b**) xy-projection of the Geant4-simulation. The dose is calculated using a voxel size identical to the one in (**a**) as the majority of energy is deposited within the first mm [see sub-figure (**e**)]. (**c**–**e**) 1D projections of the data shown in (**a**) and (**b**). In (**c**) and (**d**) the simulation has been translated so the center values of the distributions overlap. The spread of both simulation and measurement are marked with dashed lines. The center value and spread of the data were calculated excluding the outermost 1 mm in both sides (marked with a lighter blue) due to the proximity to the crystal edges.

The dose-response of LYSO:Ce was investigated by irradiating a 1 $\hbox {cm}^3$ LYSO:Ce cube under the $^{90}$Sr/$^{90}$Y-source with a constant dose rate at varying irradiation times, followed by readout using a CW laser and a PMT in photon counting mode with appropriate filters. The results are presented in Fig. 3, showing an apparent sub-linearity at doses above 2.4 Gy, which might be due to fading of the OSL signal. The dose values presented on the upper axes in this figure were estimated using Geant4-based Monte Carlo simulations to estimate the energy deposition rate, which, when scaled with the mass of the scintillator, yields the dose rate of 0.24 Gy/min. Only the mass of the first mm of the crystal was used, as the vast majority of the dose is deposited here (see simulation in Fig. 4e). The CSDA range of 1 MeV electrons, representing the expected energy of an electron emitted from the used source, in LYSO are stated to be < 1 mm in the NIST database^27^, which is in agreement with the presented simulations and further supports the approach used for estimating the dose.

### Spatially resolved 3D readout of dose using OSL

Exploiting the OSL mechanism in LYSO:Ce for post-irradiation investigation of dose deposition in 3D requires not only stimulation and detection of the number of OSL photons emitted from the crystal, but detection of where in the crystal the OSL photons were emitted. To extract this information, a novel optical readout system has been designed and constructed, capable of reading out OSL signal from OSL-based dosimeters in a layer-wise manner by stimulating OSL with a light sheet, and imaging the OSL onto a CCD camera, using appropriate filtering with an optical window between 330 and 420 nm. This allows for assessment of the number of photons emitted from a voxel volume *V* defined by the thickness of the light sheet, the system magnification, and the number of binned CCD pixels. With the interest of minimizing the readout time, a wavelength of 445 nm was chosen for the stimulating light sheet. Further description of this setup and the readout concept can be found in the “Methods” section.

Measuring the number of detected photoelectrons $N_\mathrm {pe}$ generated in each pixel allows for an assessment of the number of emitted OSL photons using the system PDE. Moreover, the number of emitted photons in a given voxel volume *V* is dependent on the deposited dose *D* and the OSL light yield as described in the previous section. Hence, the number of photoelectrons are expected to be directly proportional to both the deposited dose and the voxel volume, scaling as $N_\mathrm {pe} = (\mathrm {QE}\cdot \mathrm {LTE}\cdot \mathrm {OCE} \cdot \mathrm {LY}_\mathrm {OSL}\cdot \rho )\cdot D\cdot V,$ where $\rho$ is the sample mass density and QE and OCE are now specific for the 3D optical readout system.

Figure 4 shows a readout of the 1 $\hbox {cm}^3$ LYSO:Ce crystal irradiated for 10 min under the $^{90}$Sr/$^{90}$Y-source. The crystal was placed with the irradiated side facing away from the camera and three 1 mm wide layers were read out from the back (furthest away from the camera) to the front (closest to the camera). That is, sheet center-positions 9.5, 8.5, and 7.5 mm from the front of the sample in the given order, which from this point are referred to as layers 10, 9, and 8, respectively. Figure 4a shows the readout of layer 10 and Fig. 4b displays the result of a Geant4-simulation^26^, tracking energy deposition in the crystal step-wise during 10 ms of irradiation using the same voxel-sizes as in Fig. 4a. The dose has been calculated by scaling the simulation with the irradiation time and dividing by the mass of the voxel volume. Figure 4c–e display the projections of both simulation and measurement onto each axis. Using the distributions in the x-direction and the y-direction as probability mass functions, the centers, and spreads of the distributions were calculated, and the simulation has been translated so the centers of the two overlap. The spreads are indicated on the figures, showing a discrepancy between simulation and measurement below 10% in both directions. Note that the outermost 1 mm has been excluded in these estimations, as defects in the crystal edges scatter the stimulating light resulting in an increased signal from these volumes (see Fig. 4a along the outer perimeter). This scattering of stimulating light is possibly also the reason for the discrepancy between the simulation and measurements in Fig. 4e, as the scattered stimulating light continues readout of the occupied OSL-traps in the non-bleached layer 10 during the readout of both layers 9 and 8. The measurement presented in Fig. 4 constitute one of five repetitions of measurements made to assess any systematic errors. The voxel-wise error of the mean between these measurements was found to be below 10%, and is attributed to variations in alignments during irradiation and readout.

**Figure 5:**
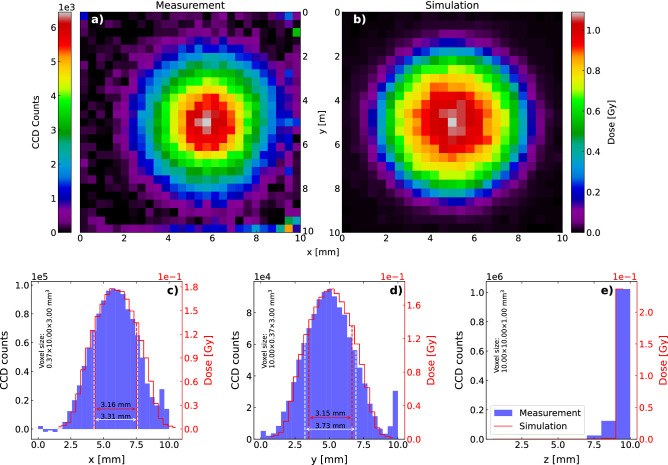
Spatially resolved 8-by-8-binned post-irradiation OSL readout from a $^{90}$Sr/$^{90}$Y-irradiated LYSO:Ce crystal. The crystal was irradiated by a $^{90}$Sr/$^{90}$Y-source for 1 min. (**a**) Readout of the first mm of the crystal, as seen from the source. The voxel size is 0.37 $\times$ 0.37 $\times$ 1.00 $\hbox {mm}^3$. For display purposes, the color map is adjusted to saturate (gray) at very high count numbers and negative values are interpreted as background fluctuations and displayed as zero counts. (**b**) xy-projection of the Geant4-simulation. The dose is calculated using a voxel size identical to the one in (**a**) as the majority of energy is deposited within the first mm [see sub-figure (**e**)]. (**c**–**e**) 1D projections of the data shown in (**a**) and (**b**). In (**c**) and (**d**) the simulation have been translated so the center values of the distributions overlap. The spread of both simulation and measurement are marked with dashed lines.

**Figure 6:**
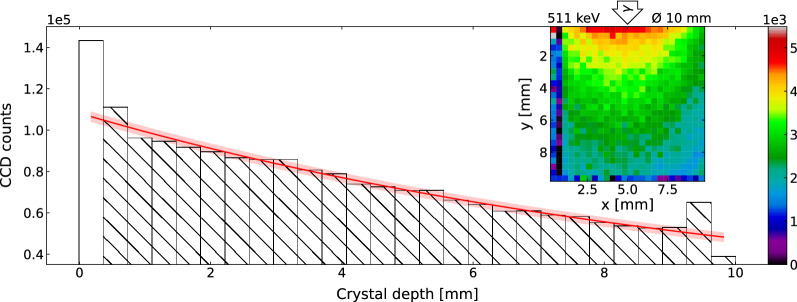
Projections of a post-irradiation 3D-readout from a $^{22}$Na-irradiated LYSO:Ce crystal. A y-projection of the 3D-readout from the central seven 1 mm wide layers of the 1 $\hbox {cm}^3$ LYSO:Ce crystal. The crystal was irradiated with a collimated Ø 10 mm 511 keV beam from a $^{22}$Na-source. A mono-exponential fit yielding an attenuation length of is $10.6\pm 0.3$ mm and corresponding uncertainties are displayed in red, where the first data point (bar without hatching) is excluded from the fitting due to the proximity to the crystal edge. The inset displays the xy-projection of the data with the arrow indicating the direction of the irradiation. Negative values are interpreted as background fluctuations and displayed as zero counts. The voxel-sizes are 0.37 $\times$ 0.37 $\times$ 7.00 $\hbox {mm}^3$ and 10.00 $\times$ 0.37 $\times$ 7.00 $\hbox {mm}^3$ for the 2D and 1D projections, respectively.

Measuring with a state-of-the-art CCD camera that is shot-noise-limited above 100 detected photoelectrons allows for assessment of the signal-to-noise ratio as $\mathrm {SNR} = \sqrt{N_\mathrm {pe}} = \sqrt{N\cdot \mathrm {CG}}$, where *N* is the number of counts and CG is the CCD conversion gain describing the number of photoelectrons per count. From the measurements in Fig. 4a acquired with $\mathrm {CG}=1.26$, we thus report an SNR of $\sqrt{800\cdot 1.26}\sim 31$ at estimated doses of $\sim$ 10 Gy in voxel-volumes of 0.046 $\times$ 0.046 $\times$ 1.00 $\hbox {mm}^3$. These results additionally enable us to express the expected number of detected photoelectrons as $N_\mathrm {pe} \approx 4.8\times 10^4 \cdot D/\mathrm {Gy}\cdot V/\mathrm {mm^3}$. In some cases an SNR of 31 may be insufficient, and hardware binning $M\times M$ pixel of the CCD before analog-to-digital conversion can effectively increase the voxel volume by a factor of $M^2$ and thus increase the SNR by a factor *M*. Figure 5 shows the results of an experiment similar to that shown in Fig. 4. In this case, the CCD was configured to bin 8 by 8 pixels before analog-to-digital conversion with $\mathrm {CG}=1.19$ and the sample was irradiated only 1 min. As expected, the spatial resolution is significantly lower in Fig. 5, however, we observe an increase in the *SNR* to $\sqrt{6000\cdot 1.19}\sim 84$ at estimated doses of only 1 Gy in voxel-volumes of 0.37 $\times$ 0.37 $\times$ 1.00 $\hbox {mm}^3$. A slight discrepancy is observed between these two measurements, as the binned readout yields an 8% higher scaling factor of $N_\mathrm {pe} \approx 5.2 \times 10^4 \cdot D/\mathrm {Gy}\cdot V/\mathrm {mm^3}$.

To illustrate the readout of irradiation deeper in the bulk of the LYSO:Ce cube, the 1 $\hbox {cm}^3$ LYSO:Ce crystal was irradiated with a Ø 10 mm collimated beam of 511 keV gamma photons from a $^{22}$Na-source. Figure 6 displays the background-subtracted results of the readout from the central seven 1-mm thick layers after this irradiation. The inset shows the 2D projection of the 3D data, illustrating the sum of all seven layers which, when further projected onto the y-axis, yields the bar-plot. This 1D-projection allows for an estimation of the attenuation length of $10.6\pm 0.3$ mm for 511 keV gamma photons in LYSO:Ce, which within two standard deviations is consistent with the literature value of 11.2 mm^28,29^. As a preliminary result, this serves as a proof-of-concept measurement illustrating the ability of the readout method to spatially resolve energy deposition using OSL. Note that this estimation was made by excluding the outermost data point due to high scattering at the crystal edge and a possible contribution from the annihilation of positrons from the source on the crystal surface.

## Discussion

The results presented above confirm the viability of using optically stimulated luminescence as the characteristic emission enabling high spatially resolved 3D dose imaging. We have demonstrated the ability to extract information about the spatial distribution of a deposited dose in an LYSO:Ce scintillator with high spatial resolution using OSL. Furthermore, we have correlated the number of photoelectrons detected when reading out from a voxel volume *V* in which a dose *D* have been deposited: $N_\mathrm {pe} \approx 5 \times 10^4 \cdot D/\mathrm {Gy}\cdot V/\mathrm {mm^3}$. Considering only statistical errors, the precision of estimated doses can be assessed from this result using the intrinsic noise in the state-of-the-art CCD camera used for readout, which is assumed to be shot-noise limited for pixel-values higher than 100 detected photoelectrons. The minimum detectable doses at the current stage in voxel volumes of 0.1 $\hbox {mm}^3$ are thus found to be $\sim \; 0.5$ Gy with a 2% precision level and $\sim \; 0.2$ Gy with a 3% precision level.

Further improvement in the readout precision is equivalent to increasing the constant of proportionality in the above expression given as $\mathrm {PDE} \cdot \mathrm {LY}_\mathrm {OSL} \cdot \rho$. With an estimated OSL yield of 100 photons per deposited MeV and a scintillator density of 7.1 g/$\hbox {cm}^3$ the PDE of the 3D readout system is estimated to be $\sim \; 1.1 \times 10^{-3}$%. The system PDE can further be theoretically estimated as the product between the quantum efficiency of the CCD ($\sim$ 80%), the light transfer efficiency in the crystal ($\sim$ 10%), and the OCE of the setup. The latter can be estimated as $\mathrm {OCE} = \frac{Tm^2}{ 4F^2(1+m)^2+m^2}$, where *T* is the transmission, which for the setup is the combined transmission of the filters and the objective ($\sim$ 40%), *m* is the magnification (0.3) and *F* is the f-number of the lens^30^. The currently used objective has an f-number of $F=4$, which yields an OCE of $3.3 \times 10^{-2}$% and consequently a PDE of $\sim \; 2.7\times 10^{-3}$% in reasonable agreement with the measured. As seen from this theoretical evaluation, the setup can be improved significantly by lowering the f-number and increasing the transmission.

Further improvements of a more technical character will be the center for further development. This includes minimization of the observed scattering of the laser sheet on damages on the crystal surfaces, which is observed to cause readout of neighboring layers. Such a minimization would significantly increase the reliability of the 3D spatial information acquired with the setup. Other challenges include characterization of the sheet profile, complete optical bleaching of the dosimeter between readouts, and consistent placement of the dosimeter in the readout setup. We stress that such technical challenges do not prohibit an assessment of the SNR provided by the system in a shot-noise limited case and that solving such problems will reduce systematic errors which in turn pushes the system towards shot-noise limitation.

Notably, all the data acquired in 3D is presented as either 2D or 1D projections, which arguably lowers the true dimensionality of the information achieved with the readout system. As evident from the z-projections in Figs. 4 and 5, 3D information is available in the readouts, but some work is still required to fully validate data acquisition from internal layers. The presented results nevertheless serve as a proof-of-concept, as the system can read out low doses with high precision and high spatial resolution over the entire length (z-direction) of the crystal, e.g. 0.5 Gy with 2% precision in voxel volumes of 0.1 $\hbox {mm}^3$.

In addition to the spatially resolved OSL measurements, we have studied both the spectral characteristics and kinetics of this photon emission process in LYSO:Ce, confirming the mechanism with the $\hbox {Ce}^{3+}$ centers as the luminescent centers. The study has been extended to estimate the OSL yield in terms of the number of photons emitted per MeV of energy deposited, resulting in an estimate on the order of 100 ph/MeV. As the OSL mechanism is competitive with the scintillation mechanism, much effort has been put into minimizing these defects in LYSO:Ce to optimize the material for TOF-PET. The presented yield is promising in this regard, as it is very low compared to both a theoretically estimated maximum OSL yield of $\sim$ 18,000 photons/MeV (see the “Methods” section) and the scintillation yield of 40,000 ph/MeV.

This OSL yield, intrinsic to the material, is linked to the achievable spatial resolution and precision as a function of deposited dose and serves as a benchmark for future material development, e.g. OSL-active nanocrystals embedded in a polymer matrix^31^. The determination of the OSL yield not only allows framing and concentrating efforts in the search for water-equivalent OSL dosimeters but enables absolute post-irradiation dose measurements without the need for external verification with validated dosimeters. Having limited the yield to a minimum of 100 OSL-photons per deposited MeV is very promising and motivates further investigations to obtain a more accurate yield based on an event-by-event readout.

We investigated the dose linearity of the OSL response in LYSO:Ce, which was observed to cover at least two orders of magnitude for irradiation with a constant and by clinical standards low dose rate of 0.24 Gy/min. As a consequence of the low dose rate and thus long irradiation time, we note that any thermally induced de-trapping of electrons in the OSL traps during irradiation could influence the response linearity. Based on the frequency factors and thermal depth of the traps found in reference 10, we estimate a lifetime of $\sim \; 200$ min at an estimated laboratory temperature of 19 $^\circ$C which potentially influences the drop in response seen at long irradiation times in Fig. 3. Hence, the dose-linearity measurements presented in Fig. 3 does not necessarily exclude dose-linearity over a wider dose range. Previous studies have demonstrated OSL response to be dose-rate independent in $\hbox {Al}_2\hbox {O}_3$:C^32,33^, but any dose-rate dependence in LYSO:Ce is subject to further investigation.

## Conclusion

We have characterized the OSL mechanism exhibited in state-of-the-art scintillator LYSO:Ce crystals in terms of spectral emission, decay kinetics, dose-linearity response, and OSL yield. Moreover, we have demonstrated the ability to image volumetric dose distributions from two different radioactive sources using the OSL photon-emission mechanism. The novel readout system enables readout of doses as low as $\sim \; 0.5$ Gy in voxel volumes of 0.1 $\hbox {mm}^3$ with a precision of 2%, making OSL a photon-emission mechanism highly relevant for 3D dose imaging. The results presented in this contribution constitute the first steps towards the validation of an OSL-based dose imaging setup in three dimensions.

## Methods

All measurements were carried out using single-crystal LYSO:Ce cubes measuring 1 $\hbox {cm}^3$ from Crystal Photonics Inc. Irradiation of this crystal was performed using either electron excitation or gamma photon and positron excitation. During and after irradiations, the crystal was kept in a dark environment, as not to empty the occupied OSL-traps. All measurements of the crystal were performed less than 10 min after irradiation to minimize any fading effects.

Electron excitation was performed using a custom made irradiation container in which samples could be position beneath a cylindrical $^{90}$Sr/$^{90}$Y-source (74 MBq in 2019) with radius 1.5 mm. The surface-to-surface distance between the crystal and source was $\sim \; 3.5$ mm during irradiations, yielding an estimated dose rate of 0.24 Gy/min to the first mm of the crystal. The medium separating the crystal and source was air.

Gamma photon and positron excitation was performed by placing the crystal on top of a steel-container holding a $^{22}$Na-source (1.85 GBq in 2011). The source was fixated within the container with direct access to the sample through a 10 cm deep cylindrical hole of 0.5 cm radius. Irradiation was performed by positioning the crystal over this hole.

### 3D optical readout

**Figure 7:**
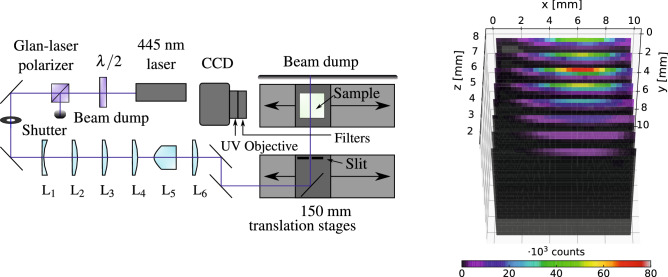
Sketch of the 3D readout-system and proof-of-concept measurement. Note that some of the components in the sketch have been rotated 90$^\circ$ around the beam for illustrative purposes. $\hbox {L}_1$: − 100 mm focal length spherical plano-concave, $\hbox {L}_2$: 175 mm focal length spherical plano-convex, $\hbox {L}_3$: 1000 mm focal length cylindrical plano-convex, $\hbox {L}_4$: 500 mm focal length cylindrical plano-convex, $\hbox {L}_5$: 30$^\circ$ Powell lens, $\hbox {L}_6$: 200 mm focal length cylindrical plano-convex. The proof-of-concept measurement was obtain by reading out a 1 $\hbox {cm}^3$ LYSO:Ce crystal irradiated under a $^{90}$Sr/$^{90}$Y-source.

The optical readout from the crystal was done using a SOPHIA-2048BR CCD camera ($13.5\times 13.5\ {\upmu }\hbox {m}^2$ pixels) in combination with a $\le$ 1 mm thick light sheet generated from the output of a laser of wavelength 445 nm. By using the laser to stimulate a specific layer of the crystal, a 2D image of that given layer was acquired with the CCD camera. Moving the sheet along the crystal provided the third dimension, and a 3D measurement was obtained by stacking the acquired 2D measurements.

All measurements reported in this contribution were acquired using the low noise quality setting of the camera with a high gain and a readout rate of 4 MHz. The conversion gain for these settings is specified by the manufacturer to be $\mathrm {CG}=1.26$ for a non-binned sensor. The conversion gain for the 8-by-8-binned sensor was assessed by measuring the change in conversion gain when binning the sensor and scaling the manufacturer’s conversion gain accordingly.

Figure 7 shows a sketch of the design of the setup. Starting from the light source, the laser beam is attenuated using a half-wave plate and a Glan-laser polarizer allowing for continuous adjustment of the intensity of the sheet without changing the laser-beam profile. The beam then passes through a shutter, that operates synchronously with the camera shutter, ensuring maximum detection of photons emitted via OSL. Next, the laser beam is magnified using a Galilean telescope before it is focused with a cylindrical plano-convex lens creating an elongated horizontal focus at the sample site. The beam additionally passes through another cylindrical plano-convex lens to adjust the vertical divergence before it reaches the Powell lens. This lens is responsible for creating an intensity-homogeneous 30$^\circ$ fan, which is then collimated by a cylindrical plano-convex lens, yielding a $\le \; 1$ mm wide $\sim \; 10$ cm high light sheet. Finally, the sheet is guided to the sample using two translation stages that move in a coordinated manner to ensure that the stimulated layer remains in focus as the object plane for the camera. Imaging the crystal layers onto the CCD was done using a Jenoptik APO macro UV-objective with appropriate filters (Edmund Optics 46-434, 84-716, and 84-720) resulting in a spectral window between 330 and 420 nm, isolating the OSL-signal from the stimulating light.

An illustrative layer-wise presentation of a proof-of-concept readout using the described setup is also shown in Fig. 7. Here, the 1 $\hbox {cm}^3$ LYSO:Ce crystal was irradiated under the $^{90}$Sr/$^{90}$Y-source with the xz-plane facing the source and electrons penetrating the crystal in the positive y-direction. The crystal was then read out in the system with the irradiated side facing upwards, with the light sheet moving in the negative z-direction of the crystal, spanning the xy-plane. The result shown in the figure represents the average of 10 repeated measurements. Compared with the readouts presented in Figs. 4 and 5, the dose distribution is now visible along the z-axis, which is the dimension provided by the moving light sheet.

### OSL photon counting readout

The OSL-based dose-response was studied using a PMT from ET Enterprises operated in photon counting mode. The experimental setup consisted of the PMT with a photocathode diameter of 25 mm, optically coupled to the sample site using a lens with a focal length of 35 mm. Appropriate filters (Thorlabs FGB37-A and Edmund Optics 84-703, 84-707, 84-717, and 15-256) providing an optical window between 330 and 420 nm were used to block the laser, which illuminated the sample at a grazing angle. A multi-channel analyzer was used to count all transistor-transistor-logic (TTL) pulses from the PMT. To avoid saturation of the PMT, neutral density filters were added to the filter set for high-dose measurements. For the 100 and 180 min irradiations, a filter with 13.3% transmission was added, and for the 1000 min irradiation, a filter with 2% transmission was added. The dark-count rate of the PMT was around 10 counts per second, yielding measurements with a large signal-to-noise ratio, where the maximum count rate was limited by the 25 ns dead-time interval between consecutive TTL pulses.

Spectrally resolved data was monitored using stimulation by three different laser diodes with wavelengths at 460 nm, 532 nm, and 664 nm, while emission was readout by a Princeton Instruments Acton SP2300 spectrometer coupled to a Pixis camera, cooled to − 75 $^{\circ }$C. The spectrometer was equipped with several filters, varying for the different stimulating wavelengths, to isolate the OSL-photons. The temporally resolved OSL data was acquired with a Ti:Sapphire 800 nm Solstice ACE femtosecond laser from SpectraPhysics, fed into a TOPAS/nirUVis optical parametric amplifier operated at a wavelength of 460 nm, which excites the OSL emission. The readout used the same ET Enterprise PMT and a FastComTech P7888 multiscaler with time-bins of 1 ns to measure photons in time-correlated single-photon-counting (TCSPC) mode. The femtosecond laser clock running at a repetition rate of 5kHz was used to trigger the TCSPC start signal, which yielded an overall instrumental response function of 6 ns FWHM. For all these studies, the LYSO:Ce crystal readout face coincided with the layer where a dose has been deposited, which differs from the geometry of the 3D readout experiments.

### OSL yield estimations

We can estimate the maximum number of electron-hole pairs available for radiative recombination in LYSO:Ce by following the relation $N_\mathrm {e-h} = \frac{E_\mathrm {dep}}{\beta E_\mathrm {bg}}$, where $E_\mathrm {dep}$ is 1 MeV of energy deposited in the material, $\beta E_\mathrm {bg}$ is the minimum energy necessary to create an electron-hole pair, and thus a photon. Considering $\beta$ = 2.3^34^, the maximum scintillating light yield of LYSO:Ce would range around 58,000 photons per MeV if all created electron-hole pairs will recombine radiatively. This assumes that the energy transfer efficiency to the luminescent centers, *S* and their quantum efficiency QE equals unity. However, until now intrinsic light yields of LYSO:Ce scintillators superior to 40,000 ph/MeV have not been reported^5^. This sets an upper limit on the number of OSL photons that could be harvested in LYSO:Ce to be around 18,000 ph/MeV, which is the difference between maximum photon conversion efficiency and the intrinsic scintillation yield assuming $1-S$ to be the efficiency of energy transfer to the OSL traps.

The OCE of the experimental setup, i.e. the fraction between the total number of photons at the sample position over the total number of photons arriving at the PMT photocathode, was measured by monitoring the afterglow of $\hbox {Al}_{{2}}\hbox {O}_{{3}}$ single crystals, a dosimeter with a radioluminescence spectrum in the visible region. We compared the number of counts when measuring in direct contact with the window in front of the PMT photocathode and when replacing the LYSO:Ce crystal in the OSL readout setup. This method allowed for estimation of the OCE of our setup in relation to a known and studied air-coupled configuration with reported light-transfer efficiency values^5^. The choice for this method was made to avoid any counting saturation arising from TTL pulses arriving too closely in time, i.e. the probability of having only one photon arriving at the PMT photocathode within the 25 ns dead time of the detector is maximized. For this reason, we chose a slow photon emission process such as afterglow in $\hbox {Al}_{{2}}\hbox {O}_{{3}}$ over scintillation due to self-activation in LYSO:Ce to account for the OCE of the setup.

### Geant4-based Monte Carlo simulations

The Geant4 simulations presented in this work were performed using the Geant4.10.07 distribution and the FTPT_BERT physics list. Results using such code have previously been validated^5,35^ by comparing to experimental data. The scoring volume was defined to be a 10 $\times$ 10 $\times$ 10 $\hbox {mm}^3$ cube of undoped lutetium orthosilicate, which for simulating purposes replicate LYSO:Ce. The main parts of the internal geometry of the irradiation setup were included in the simulation to mimic the actual irradiation profile. The electrons were generated using the $^{90}$Sr/$^{90}$Y-spectrum from the literature^36^. The source itself was a disk of radius 1.5 mm with an isotropic angular profile located 3.5 mm from the surface of the scoring volume. The step-wise energy deposition and post-step coordinates of each particle depositing energy in the scoring volume were saved and the 3D energy deposition matrix was generated through post-processing in Python.

## Data Availability

The datasets generated and/or analysed during the current study are available in the Mendeley Data repository. DOI: 10.17632/gnvxmbwkpd.1.
